# Increased incidence of motor neuron disease in Sweden: a population-based study during 2002–2021

**DOI:** 10.1007/s00415-024-12219-1

**Published:** 2024-02-22

**Authors:** Sofia Imrell, Fang Fang, Caroline Ingre, Stefan Sennfält

**Affiliations:** 1 Department of Clinical Neuroscience, Karolinska Institute, Stockholm, Sweden; 2 Department of Neurology, Faculty of Medicine and Health, Örebro University, Örebro, Sweden; 3 Unit of Integrative Epidemiology, Institute of Environmental Medicine, Karolinska Institute, Stockholm, Sweden; 4 Department of Neurology, Karolinska University Hospital, Stockholm, Sweden

**Keywords:** Motor neuron disease, Amyotrophic lateral sclerosis, Incidence, Epidemiology

## Abstract

**Background:**

Motor neuron diseases (MND), with amyotrophic lateral sclerosis constituting most cases, are rare conditions of unknown etiology. There have been reports of an increase in incidence during the latter half of the twentieth century in various Western countries, including Sweden. This study provides updated data on the incidence of MND in Sweden during the last 20 years.

**Methods:**

Data was obtained from the Swedish National Patient Register on individuals diagnosed with MND from 2002 to 2021 and analysed in relation to group level data for the entire Swedish population. Incidence rates were calculated and presented in relation to year, age, sex, and region.

**Results:**

In the early 2000s, there was a crude incidence rate of 3.5–3.7 per 100,000 person-years, which then increased to 4.0–4.6 from 2008 onward. Age standardization to the starting year (2002) partially mitigated this increase. The incidence rate was greater among men compared to women and was highest within the age range of 70 to 84 years. There were indications of a higher incidence rate in the northernmost parts of the country, although the difference was not statistically significant.

**Conclusions:**

The incidence rate of MND in Sweden now seems to have surpassed 4 cases per 100,000 person-years. This is higher when compared to both other European countries and previous Swedish studies. It remains to be determined if this increase reflects an actual increasing incidence of MND in Sweden or is due to other factors such as better registry coverage.

**Supplementary Information:**

The online version contains supplementary material available at 10.1007/s00415-024-12219-1.

## Introduction

Motor neuron diseases (MND) are rare conditions characterized by motor neuron dysfunction and death. Although the group also includes other similar diseases such as primary lateral sclerosis (PLS) [1] and progressive spinal muscular atrophy (PSMA), [2] it is primarily comprised of Amyotrophic lateral sclerosis (ALS) [3] which is the focus of the present paper and most previous literature on MND epidemiology. ALS rapidly progresses from focal motor weakness to respiratory failure, resulting in death, often within approximately 2–4 years from the onset of symptoms [4]. Consequently, the prevalence of ALS is typically 2–3 times the incidence rate, except in Asian populations where patients tend to experience on average a slower disease progression [5]. While most research has focused on populations of European descent, there appears to be significant geographical variation in the global incidence rate of ALS. A recent review reported an incidence rate of approximately 2.31 per 100,000 person-years in Europe, 2.35 in North America, 1.25 in Latin America, 0.93 in Asian countries (excluding Japan), and 1.76 in Japan [5]. The 2016 global disease burden reported an incidence rate of 2.0 per 100,000 person-years for Western Europe [6]. The incidence of ALS also varies greatly within Europe. Research from the last two decades has indicated an incidence rate of up to 4 to 6 per 100,000 person-years in some regions within the Nordic countries, England, and Italy, [7–11] whilst figures from other regions are more in line with the European estimates cited above [12–17]. A significant increase in the incidence rate of ALS during the latter half of the twentieth century has also been reported in various Western countries, [18] including Norway, [19] Finland, [20] Scotland, [21] and New Zeeland, [22] which is unlikely solely attributed to an ageing population. A similar trend has been observed in Sweden, showing an increase in age-standardized incidence rate of ALS from 2.32 to 2.98 per 100,000 person-years from 1991 to 2005. [23]

Regularly tracking trends in MND incidence is crucial for improving healthcare planning. Additionally, since the underlying causes of MND remain unidentified for most patients, regularly updated data on disease incidence can be instrumental in shedding light on factors potentially linked to the onset of the disease. The present study therefore aims to provide updated data on the incidence of MND during the last 20 years in Sweden in relation to demographic and geographic variables.

## Methods

### Swedish population

The study encompasses the entire Swedish population during the years 2002 to 2021, representing the population at risk. Data at group level for the Swedish population within this period was acquired from Statistics Sweden, including population figures per year, region, age group, and sex. In 2002, Sweden's population was approximately 8,930,000, and by 2021, it had increased to 10,440,000. The population is predominantly concentrated in the southern and central regions of the country, while the northern parts are relatively sparsely populated.

### MND patients

Data on individuals newly diagnosed with MND from 2002 to 2021 was obtained from the Swedish National Patient Register (SNPR). MND patients were identified using the ICD-10 code G12.2 with ALS expected to account for most, approximately 90%, of cases [1, 2]. However, since the code also includes other conditions such as PLS and PSMA and does not specify the exact diagnosis we chose to use the term MND. We obtained de-identified data from the SNPR, including year of birth, sex, and region of residency within Sweden. We identified MND patients at the initial diagnosis in relation to a hospital visit, where MND was either registered as the primary diagnosis for the visit or as a secondary diagnosis.

The SNPR was established in 1964 and, as of 1987, included more than 99% of all somatic and psychiatric hospital discharges in Sweden, with a high validity for most diagnoses [24]. Since 2001, hospital-based outpatient physician visits are also registered in this register, however, coverage of outpatient contacts is approximately 80%. The discrepancy is mostly accounted for by lower registration from private caregivers [24].

We also used a second data source to compare estimated incidence rates and explore the composition of diagnoses within the ICD-10 code G12.2 in the study population. The Swedish Motor Neuron Disease Quality Registry (SMNDR) has been shown to have a good coverage of MND patients from the Stockholm region since 2017 and is completed by the treating medical staff of the MND patients, primarily neurologists [25]. We obtained data from this registry including all MND patients diagnosed in the Stockholm region between 2017 and 2021, including age, sex and the exact diagnosis (ALS, PLS, PSMA, and Kennedy disease).

### Statistical analysis

Categorical variables were summarized as proportions (percent) and age at diagnosis was reported as median together with the 25th to 75th percentile (interquartile range [IQR]). Incidence rates were rounded to one decimal.

Due to an increasing mean age in the Swedish population during the study period (40.57 in 2002, 41.18 in 2012, and 41.56 in 2021), the age- and year-specific incidence rates were standardized to the age distribution of the starting year, i.e., 2002. Specifically, using the proportional distribution of inhabitants in each five-year age group in the year 2002 as the standard, we calculated the age-standardized incidence rates of MND at subsequent years. We also used the 1990 Swedish population as the standard, to allow for a comparison with a previous study [23].

The average regional incidence rate during the study period was calculated by taking the average yearly number of cases (for each region) during 2002–2021 divided by the number of inhabitants as of 2012 (roughly the middle of the study period). We employed the Chi-squared test to investigate the statistical significance of disparities among regions. The expected number of cases in each region was determined by multiplying the national incidence rates with the regional population sizes, under the null hypothesis that the incidence rates were uniform across regions. We made a comparison between all individual regions as well as between larger areas, namely the southern, central, and northern parts of Sweden.

Finally, we compared all MND patients registered in the SNPR versus the SMNDR during 2017–2021, to compare estimated incidence rates and to understand the composition of different MND diagnoses within the ICD-10 code G12.2.

## Results

### Patient characteristics

**Fig. 1:**
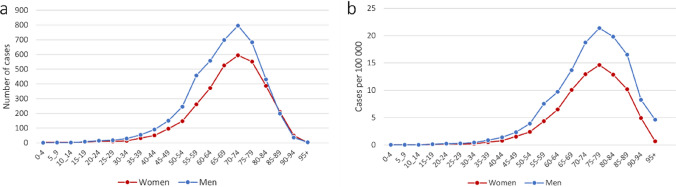
The number of cases (**a**) and incidence rate (**b**) of MND during 2002–2021 in Sweden by age and sex. Incidence rates were not age standardized

The total number of newly diagnosed MND cases in Sweden was 7,805 during 2002–2021, including 4,477 (57.4%) males and 3,328 (42.6%) females. The distribution of cases exhibited a pronounced bias toward older individuals (Fig. 1a). The median age at diagnosis was 70 years (61–77): 69 (60–76) for men and 71 (62–78) for women. The mean age at diagnosis was 68 years overall.

### Incidence rate

**Fig. 2:**
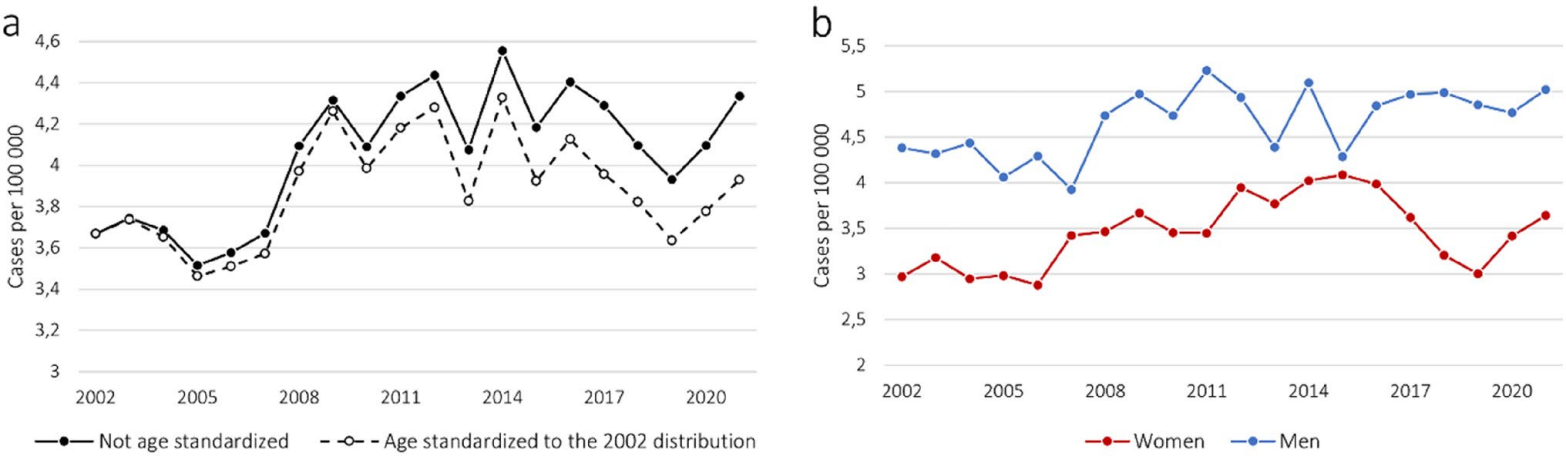
Incidence rate of MND during 2002–2021 in Sweden. With or without age standardization to the age distribution of 2002 Swedish population (**a**) and by sex (**b**, not age-standardized)

During the whole study period, the average crude incidence rate of MND was 4.1 per 100,000 person-years. In the early 2000s, there was an incidence rate of 3.5 to 3.7 per 100,000 person-years, which then increased to 4.0 to 4.6 per 100,000 person-years from 2008 onward (Fig. 2a, Supplementary Table 1). When we applied age standardization using the reference year 2002, the observed increase in the incidence rate mitigated to some extent. Similar results were noted when we used the reference year 1990 (Supplementary Fig. 1).

### Age and sex

The crude incidence rate of MND was greater among men compared to women, with an incidence rate of 3.5 for women and 4.7 for men, per 100,000 person-years, over the entire study period (Fig. 2b, Supplementary Table 1). The highest incidence rate was observed within the age range of 70 to 84 years, a pattern consistent for both sexes (Fig. 1b). Of note, a slight decline was observed towards the latter part of the study period, however only among women.

### Geographical variation

**Fig. 3:**
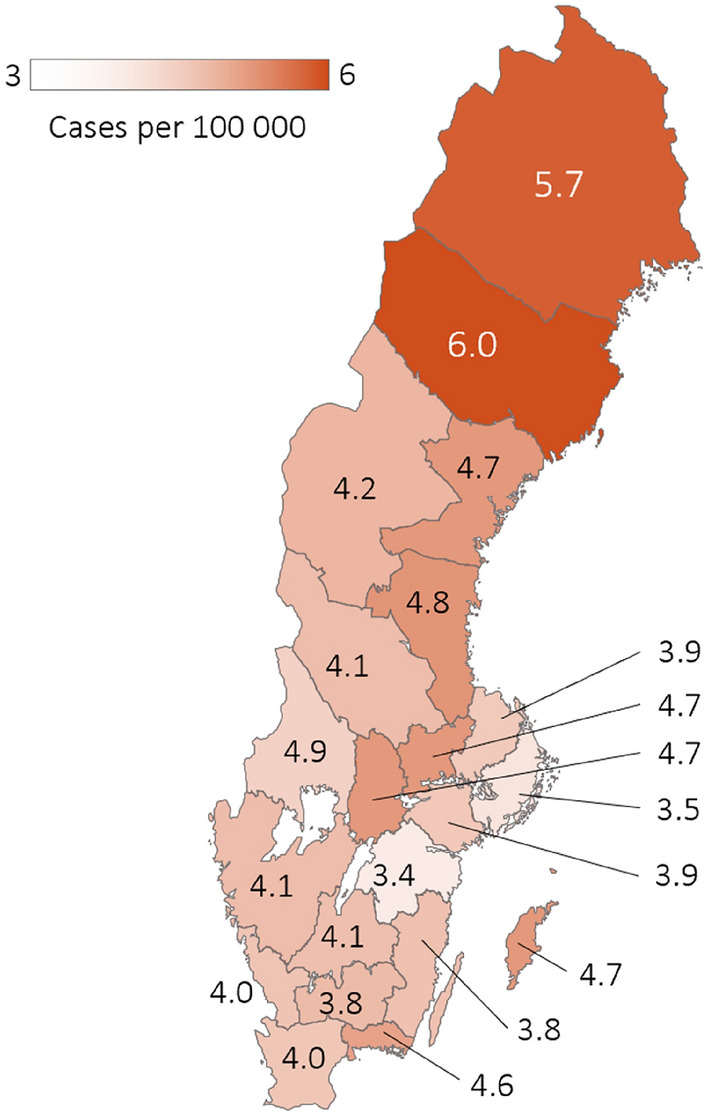
Average regional incidence rates of MND during 2002–2021 in Sweden. Incidence rates were rounded to one decimal and not age standardized

The average incidence rate of MND during 2002–2021 was higher in the northern part of Sweden, notably in the Västerbotten and Norrbotten regions (farthest to the north), where the crude incidence rate approached 6 per 100,000 person-years (Fig. 3, Supplementary Table 2). When looking at larger areas, we observed that, for the southern, central, and northern parts of the country, the incidence rates were 4.0, 3.9, and 5.2, per 100,000 person-years, respectively (Supplementary Table 3). Upon formal testing there was however no statistical difference between individual regions (p = 0.98) nor between larger areas (southern, central, and northern parts of Sweden) (p = 0.08).

Of note, the regions with the highest incidence rates also exhibited a slightly higher average age of the general population. As of 2012, the mean age in Västerbotten and Norrbotten was 41.5 and 43.5, respectively, as compared to the national mean of 41.2, years.

### Comparison to a second data source

During 2017–2021, there were 367 new cases of MND in the Stockholm region registered in the SMNDR (Supplementary Table 4), compared to the 409 MND patients registered in the SNPR (i.e., 89.7%). Sex distribution was similar in these two datasets (41.6% versus 43.3% males). Among the 367 cases in the SMNDR, there were 328 cases of pure ALS (89.4%), 16 cases of PLS (4.4%), 22 cases of PSMA (6.0%), and 1 case of Kennedy disease (0.3%).

## Discussion

In this paper, MND patients were identified using the ICD-10 code G12.2, primarily comprised of ALS. Since the code also included other conditions such as PLS or PSMA we chose to use the term MND. However, it is debated whether PSMA and PLS are separate entities or part of the ALS spectrum [1, 2], and some previous epidemiological research refers to the whole group as ALS. Also, analysis of a second data source including differentiation within the MND group suggested that approximately 90% of all MND patients were originally diagnosed as ALS, similar to previous reports [1, 2]. We therefore focus our discussion on ALS, but when comparing our results to previous reports, it should be noted that our estimations may be somewhat higher compared with studies only considering pure ALS.

Our findings showed an average crude incidence rate of 4.1 per 100,000 person-years for MND, during 2002–2021 in Sweden: 3.5 for females and 4.7 for males. This is a higher estimate compared to the previously reported European average of 2.0–2.31 per 100,000 person-years for ALS [5, 6]. This difference might partly be explained by better detection of MND patients due to the high-quality patient registration of the Swedish health care system but may also reflect a true higher incidence of MND in Sweden compared to other European countries. However, recent reports from some regions in other Nordic countries, England, and Italy have shown an incidence rate of ALS of 4 to 6 per 100,000 person-years [7–11]. An increasing south-to-north gradient in the incidence rate of MND has been shown in studies from several European countries [26]. This was suggested in our study as well as a previous Swedish study, [23] but was not statistically significant in either of these.

During the study period spanning from 2002 to 2021, there was an escalation in the incidence rate of MND in Sweden, rising from 3.5–3.7 to over 4 per 100,000 person-years. A notable portion of this increase might be attributed to demographic shifts, given that the mean age in Sweden has steadily risen over the last two decades, from 40.6 in 2002 to 41.6 in 2021. Nevertheless, even after adjusting for age, a slight upward trend in incidence remained observable. These findings align with a broader trend of rising MND incidence observed in multiple Western countries since the mid-twentieth century [18–22]. Importantly, since most studies have conducted age-standardized analyses, the increase cannot be solely attributed to an aging population. Specifically, the results of the present study demonstrate a continued increase in MND incidence in relation to a previous Swedish study where we reported an increase from 2.32 to 2.98 per 100 000 person-years from 1991 to 2005 after age standardization by the 1990 Swedish population (mean age 39.42) [23]. As the mean age was higher, 40.6, in the 2002 Swedish population, we, in a sensitivity analysis, standardized the incidence rates of MND during 2002–2021 to the age distribution of the 1990 Swedish population, and found that, although the estimated incidence rates diminished to some extent, they were still above the levels observed in the previous study. An additional explanation for the higher incidence rates observed in the present study, compared to the previous study, might be the inclusion of outpatient hospital data in the SNPR from 2001 onward which might have allowed for more cases to be identified in the present study with a more recent study period. However, as the vast majority of MND patients will eventually be admitted for inpatient care at some point during the care process for MND, this add-on might be of limited impact.

A certain year-to-year variation in incidence rate can be anticipated in a rare diagnosis such as MND. Nevertheless, the slight decline observed towards the latter part of the study period in the present study may partially be attributed to a significant societal event, namely the COVID-19 pandemic. During this period, there was a decrease in healthcare utilization [27]. While the impact of the pandemic on the diagnostics of MND has not been studied much, it is possible that it had a negative effect. However, as a similar trend was also evident in 2018 and 2019, predating the onset of the pandemic, alternative explanations might be at play. Of note, the reduction in incidence rate during these years was only observed among women.

In summary, if the findings in this study represent an actual increase in the incidence of MND, further research is warranted to delineate the causes for this, considering both genetic and environmental factors. First, there is significant variability in the occurrence of mutations in key ALS-related genes among different ethnic groups [28]. Specifically for the Nordic countries, there is a higher occurrence of hexanucleotide expansion mutations in the *C9orf72* gene, and a geographical gradient (more prevalent in the northern regions) has been observed in Finnish and Norwegian populations [29, 30]. However, the specific distribution of these mutations within Sweden remains unknown. Regarding environmental factors, although there has been extensive research in this area, only a limited number of factors have emerged as credible candidates, with smoking being the only one established [31]. Also, an important factor to consider is the specific age groups where an increase was noted. However, the mean age at diagnosis observed in the present study, i.e., 68 years, is similar to that observed in the previous Swedish study with a study period of 1991–2005 [23].

### Methodological considerations

The SNPR, which encompasses both inpatient and outpatient hospital visit data, has extensive coverage, making it highly improbable that a significant portion of diagnosed MND patients would go unregistered. While it is conceivable that a small number of patients may have never sought specialized healthcare for MND, this is expected to be a rare occurrence. Indeed, a comparison of the Stockholm region between SNPR and the SMNDR, the latter considered to be reliable in this region, showed a larger number of cases in the SNPR. Reasons might be that some of the MND patients identified in the SNPR did not turn out to have MND (e.g., wrong initial diagnosis or changed diagnosis during the disease course) or that the SMNDR is not 100% complete for the Stockholm region as it is reliant on manual input for each patient from the treating medical staff.

Another consideration is the potential underdiagnosis of MND in general. However, despite the relatively lengthy diagnostic delay in Sweden, which averages around one year, [32] the majority of patients are likely to eventually receive a diagnosis during the course of their illness. Sweden's universal healthcare system is characterized by minimal barriers to access, further alleviating concern of underdiagnosis in MND due to disparities related to sociodemographic factors. However, we did not have access to individual-level information on sociodemographic and lifestyle factors, precluding the examination of incidence rates in relation to educational level, occupation, smoking habits, etc.

## Conclusion

We report a continued increase in the incidence rate of MND in Sweden during the last two decades. While an aging population contributed, it did not account for the entire increase. The influence of genetic and environmental factors remains to be determined. To gain further insights in the temporal trend of MND incidence, it is essential to obtain high-quality epidemiological data from various regions worldwide. Ideally, such studies should encompass genetic testing as well.

### Supplementary Information

Below is the link to the electronic supplementary material.

Supplementary file1 (PDF 324 KB)

## Data Availability

The data that support the findings of this study are available on request from the corresponding author after obtaining the appropriate ethical approval. The data are not publicly available due to privacy or ethical restrictions.
