# Supplementary material for: Increased incidence of motor neuron disease in Sweden: a population-based study during 2002–2021
Source: J Neurol. 2024 Feb 22;271(5):2730–5. doi: 10.1007/s00415-024-12219-1 (PMC11055737; doi:10.1007/s00415-024-12219-1)
Supplement: Supplementary file 1 — Supplementary file1 (PDF 324 KB) [file 415_2024_12219_MOESM1_ESM.pdf]

Supplementary material

Increased incidence of motor neuron disease in Sweden: a population-based study during 2002-2021

Supplementary Table 1 Summary table of cases of motor neuron disease per age group and sex. Aggregated for all regions and the entire study period of 2002-2021

	Age group																				
	0-4	5-9	10-14	15-19	20-24	25-29	30-34	35-39	40-44	45-49	50-54	55-59	60-64	65-69	70-74	75-79	80-84	85-89	90-94	95+	Total
**Men**																					
100 000 person-years	55.24	56.04	57.58	59.43	61.32	64.12	64.36	64.82	65.21	64.50	62.63	60.89	57.43	50.98	42.51	31.89	21.69	12.00	4.35	0.87	957.85
Cases	1	2	2	8	15	18	29	55	91	150	245	458	558	698	797	682	430	198	36	4	4477
Cases per 100 000 person-years	0.02	0.04	0.03	0.13	0.24	0.28	0.45	0.85	1.40	2.33	3.91	7.52	9.72	13.69	18.75	21.39	19.83	16.51	8.28	4.62	4.67
**Women**																					
100 000 person-years	52.25	53.07	54.54	55.69	57.64	61.07	61.50	62.15	62.85	62.50	61.09	60.03	57.35	52.28	45.84	37.72	30.02	20.63	9.95	2.96	961.11
Cases	3	3	3	5	12	10	14	32	50	96	147	261	373	526	594	552	386	210	49	2	3328
Cases per 100 000 person-years	0.06	0.06	0.06	0.09	0.21	0.16	0.23	0.51	0.80	1.54	2.41	4.35	6.50	10.06	12.96	14.63	12.86	10.18	4.92	0.68	3.46
**Total**																					
100 000 person-years	107.49	109.10	112.13	115.12	118.96	125.19	125.85	126.97	128.05	127.00	123.72	120.92	114.78	103.25	88.35	69.61	51.71	32.62	14.30	3.83	1918.96
Cases	4	5	5	13	27	28	43	87	141	246	392	719	931	1224	1391	1234	816	408	85	6	7805
Cases per 100 000 person-years	0.04	0.05	0.04	0.11	0.22	0.22	0.34	0.68	1.10	1.95	3.18	5.96	8.09	11.75	15.69	17.72	15.82	12.46	5.81	1.62	4.07

Supplementary Table 2 Summary table of regional incidence rates of motor neuron disease during 2002-2021. The chi-squared test analysing the statistical significance of the difference yielded a p-value of 0.98

	Region, south-north																				
	Skåne	Blekinge	Kronoberg	Halland	Kalmar	Jönköping	Gotland	Västra Götaland	Östergötland	Värmland	Örebro	Södermanland	Västmanland	Stockholm	Uppsala	Dalarna	Gävleborg	Jämtland	Västernorrland	Västerbotten	Norrbotten
Aggregated greater area	South	South	South	South	South	South	South	South	South	Central	Central	Central	Central	Central	Central	Central	North	North	North	North	North
100 000 inhabitants in 2012	12.62	1.52	1.86	3.04	2.33	3.39	0.57	15.99	4.33	2.73	2.83	2.74	2.56	21.23	3.41	2.76	2.76	1.26	2.42	2.60	2.49
Average number of cases per year	50.1	6.95	7.1	12.15	8.75	13.85	2.7	65.75	14.65	13.65	13.35	10.65	12.1	73.85	13.15	11.35	13.15	5.35	11.35	15.55	14.1
Average incidence rate	3.97	4.56	3.82	4.00	3.75	4.09	4.71	4.11	3.38	5.00	4.72	3.88	4.73	3.48	3.85	4.11	4.76	4.24	4.69	5.98	5.67
Expected number of cases bases on the average incidence rate for all regions (4.36)	55.0	6.6	8.1	13.2	10.2	14.8	2.5	69.7	18.9	11.9	12.3	11.9	11.2	92.5	14.9	12.0	12.0	5.5	10.5	11.3	10.8

Supplementary Table 3 Summary table of aggregated greater area incidence rates of motor neuron disease. The chi-square test analysing the statistical significance of the difference yielded a p-value of 0.08

	Aggregated greater area		
	South	Central	North
100 000 inhabitants in 2012	45.65	38.26	11.53
Average number of cases per year	182	148.1	59.5
Average incidence rate	3.99	3.87	5.16
Expected number of cases bases on the average incidence rate for all aggregated greater areas (4.34)	198	166	50

Supplementary Table 4 Comparison between motor neuron disease case numbers in the SNPR and the SMND in the Stockholm region during 2017-2021

	SNPR, including ICD-10 G12.2.			SMNDR, including ALS, PLS, PSMA, and Kennedy disease.		
	Men	Women	Total	Men	Women	Total
2017	41	28	69	43	34	77
2018	45	30	75	38	26	64
2019	43	33	76	36	26	62
2020	50	43	93	42	37	79
2021	60	36	96	49	36	85
Total (%)	239 (58.4)	170 (41.6)	409	208 (56.7)	159 (43.3)	367

SNPR= Swedish National Patient Register
SMNDR=Swedish Motor Neuron Disease Quality Register

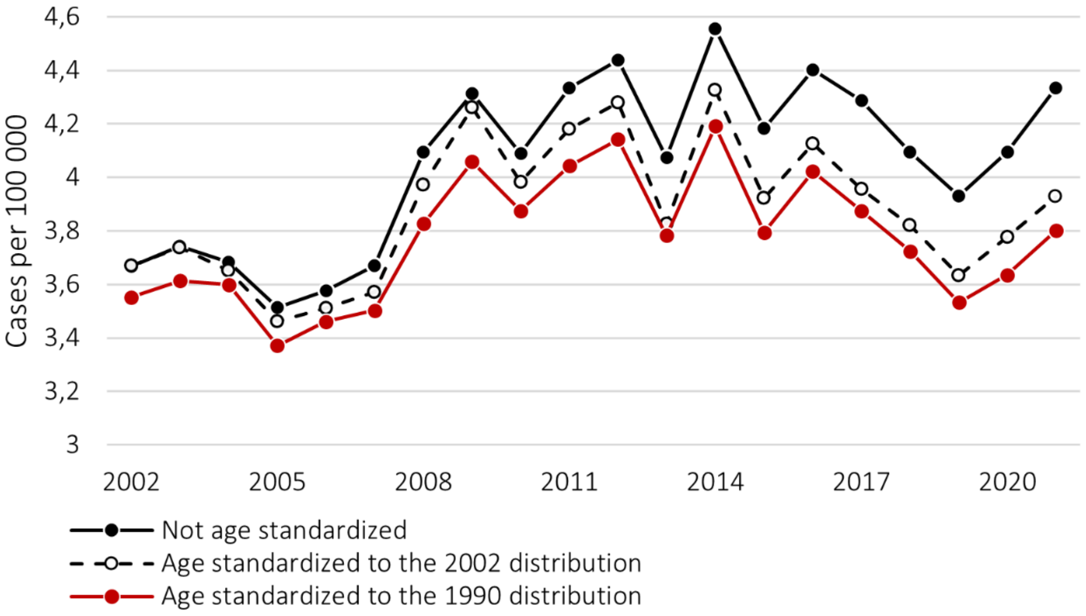

Supplementary Fig. 1 Reproduction of Fig. 2a with the addition of age standardization to the age distribution of the 1990 Swedish population
